# Exploring the anti-cancer and antimetastatic effect of Silymarin against lung cancer

**DOI:** 10.1016/j.toxrep.2024.101746

**Published:** 2024-09-28

**Authors:** Srithika Srinivasan, Aruchamy Mohanprasanth, Ahmed Nadeem, Muthupandian Saravanan

**Affiliations:** a Saveetha Medical College, Saveetha Institute of Medical and Technical Science (SIMATS), Chennai, India; b AMR and Nanotherapeutics Lab, Department of Pharmacology, Saveetha Dental College and Hospital, Saveetha Institute of Medical and Technical Science (SIMATS), Chennai, India; c Department of Pharmacology and Toxicology, College of Pharmacy, king Saud University, Riyadh, Saudi Arabia

**Keywords:** Silymarin, Lung cancer, Metastasis, Matrix metalloprotease, Antioxidant

## Abstract

Lung cancer metastasis remains a significant challenge in cancer therapy, necessitating the exploration of novel treatment modalities. Silymarin, a natural compound derived from milk thistle, has demonstrated promising anticancer properties. This work explored the inhibitory effects of silymarin on lung cancer metastasis and revealed the underlying processes, focusing on matrix metalloproteinase (MMP) 2 and MMP-9 activities. Using a combination of in vitro and molecular docking analyses, we found that silymarin effectively reducing the lung cancer cells' motility and invasion by modulation of expression of MMP-2 and MMP-9. Furthermore, MTT assays revealed a dose-dependent inhibition of cell proliferation upon silymarin treatment and found the IC_50_ value at 58 μM. We observe that apoptotic morphology characteristic in silymarin treated groups. Cell cycle analysis exhibit the cell cycle arrest at G1 phase, 25.8 % increased apoptosis in silymarin treated groups, as evidenced by Annexin V staining. Moreover, silymarin treatment shows the lipid peroxidation in elevated level and reduced in enzymatic antioxidant level, indicating its potential role in mitigating oxidative stress induce cell death. Gelatin zymography assay indicates the silymarin has ability to inhibit the MMP-2 and MMP-9 expression in lung cancer. Additionally, cell migration assays and colony formation assays demonstrated impaired migratory and colony-forming abilities of lung cancer cells when treated with silymarin. Molecular docking studies further supported the binding affinity of silymarin with MMP-2 and MMP-9, demonstrate the −10.26 and −6.69 kcal/mol of binding energy. Collectively, our findings highlight the multifaceted anticancer properties of silymarin against lung cancer metastasis, providing insights into its therapeutic potential as an adjuvant treatment strategy.

## 1 Introduction

Lung cancer represents a profound threat to public health, persistently standing as one of the foremost causes of cancer-induced mortality on a global scale. Lung cancer has a terrible impact on lives lost; it claims more lives annually than breast, prostate, and colorectal cancers combined. With a five-year survival rate of fewer than 18 %, the disease is marked by its high mortality rate and aggressive nature [1]. Lung cancer is frequently detected at an advanced stage, when there are few alternatives for curative treatment, which contributes significantly to the high death rate [2]. Among the different types of lung cancer, non-small cell lung cancer (NSCLC) is the predominant form, making up around 85 % of all occurrences. NSCLC is further classified into several subtypes, with adenocarcinoma being the most prevalent. Adenocarcinoma arises from the glandular cells of the lung and is often found in the outer regions of the lungs. Other types of NSCLC are squamous cell carcinoma, which begins in the squamous cells that line the airways, and large cell carcinoma, which can develop in any lung region and typically grows and spreads faster than other NSCLC subtypes [1].

Although there have been notable advancements in treatment options like surgery, chemotherapy, radiotherapy, and targeted therapies, the prognosis for lung cancer patients remains poor, as the disease is still largely incurable. For early-stage lung cancer, surgical intervention is often the favored treatment approach; however, many patients are diagnosed at a stage where the tumor is inoperable. Chemotherapy and radiotherapy are standard treatments for advanced lung cancer, but their effectiveness is often limited by resistance mechanisms and significant side effects [3]. In recent years, targeted therapies, which specifically inhibit molecular pathways involved in tumor growth and progression, have shown promise in treating certain subsets of lung cancer. For example, drug targeted at mutations in the epidermal growth factor receptor (EGFR) or alterations in the anaplastic lymphoma kinase (ALK) gene have greatly enhanced the prognosis for patients possessing these particular genetic changes. [4]. However, these therapies are only effective in a minority of patients and resistance often develops over time.

One major factor contributing to the poor prognosis for lung cancer patients is metastasis, which accounts for most cancer-related fatalities. Metastasis involves cancer cells migrating from the initial tumor site to other parts of the body, forming secondary tumors that are typically more challenging to manage. The metastatic spread of lung cancer cells can occur through the bloodstream or the lymphatic system, allowing the cells to colonize vital organs such as the brain, liver, and bones [5]. Once metastasis has occurred, the disease becomes much more challenging to control, and treatment options become largely palliative rather than curative. Understanding mechanisms that drive metastasis is essential for creating successful therapeutic approaches to prevent this process. Metastasis consists of a multifaceted sequence of steps: local invasion of nearby tissues, entry into the bloodstream or lymphatic system (intravasation), survival during circulation, exit from the vessels into distant tissues (extravasation), and establishment in new locations. Every stage in this process is controlled by numerous molecular signals and crosstalk between the cancer cells and their microenvironment [6]. Key players in this process include matrix metalloproteases (MMPs), which degrade the extracellular matrix and basement membranes, facilitating cancer cell invasion and migration. A crucial component of this process involves the breakdown of the extracellular matrix (ECM), which aids in tumor cell invasion and migration [7]. Matrix metalloproteases (MMPs), especially MMP-2 and MMP-9, are vital for ECM degradation. These MMPs are a group of zinc-dependent enzymes essential for remodeling the ECM [8]. Among the different MMPs, MMP-2 (gelatinase A) and MMP-9 (gelatinase B) are notably significant in cancer metastasis due to their potential to degrade type IV collagen, a primary element of the basement membrane. The degradation of the basement membrane is a pivotal step in cancer cell invasion and migration. [9]. The complexity of MMP activity regulation encompasses transcriptional oversight, activation of dormant proenzymes, and inhibition through tissue inhibitors of metalloproteases (TIMPs) [10]. Dysregulation of MMP expression and activity is a hallmark of cancer progression and metastasis. Elevated levels of MMP-2 and MMP-9 potential corelated in increased invasive and metastatic potential in various cancers, including lung cancer [11]. Therefore, targeting MMPs, specifically MMP-2 and MMP-9, represents a promising new therapeutics avenue for inhibiting cancer metastasis.

Silymarin, derived from the seeds of Silybum marianum (milk thistle), is notable for its wide range of pharmacological benefits, such as anti-inflammatory and antioxidant effects, attracting considerable interest [12]. Silymarin has been traditionally used for its hepatoprotective properties, but recent studies have highlighted its potential anticancer effects [13]. The antimetastatic properties of silymarin are of particular interest, as it has been demonstrated to influence several molecular pathways involved in cancer progression and metastasis. The primary objective of this study to investigate the impact of silymarin on the proliferation of lung cancer A549 cells, particularly by examining its impact on the expression and activity of MMP-2 and MMP-9. This study might open avenues for crafting innovative therapeutic approaches aimed at tackling lung cancer metastasis, ultimately leading to enhanced patient outcomes.

## 2 Material and methods

### 2.1 Materials

Silymarin [CatLog No: S0292], procured from sigma Aldrich, USA. DMEM, Fetal Bovine Serum, Antibiotics solution, 1×0.25 % Trypsin are purchased from Gibco. Propidium Iodide and FITC Annexin V/Dead cell Apoptosis Kit was acquired from Invitrogen, USA. All other chemical is obtained from local vendor, India in analytical grade.

### 2.2 Methods

#### 2.2.1 Prognostic characteristics of MMP −2 and −9

To assess the prognostic significance of Matrix metalloprotease −2 and −9 genes, we employed the mRNA expression levels of MMP-2 and −9, as well as their metastatic potential in Lung adenocarcinoma, which were examined using data sourced from the UALCAN database (http://ualcan.path.uab.edu/) [14], [15]. Further, we analysis the overall survival using GEPIA database (http://gepia.cancer-pku.cn/).

#### 2.2.2 Cell culture

The human non-small cell lung cancer (NSCLC) cell line, A549, was obtained from the National Centre for Cell Sciences (NCCS) in Pune, India. The cells were cultured in a CO_2_ incubator, supplemented with 10 % fetal bovine serum (FBS), 1 % penicillin-streptomycin antibiotic combination, and DMEM medium.

#### 2.2.3 Evaluate the cytotoxicity effect of silymarin in Lung cancer A549 cell line

5000 A549 lung cancer cells were placed into each well of a 96-well plate and allowed to grow until they reached 70 % confluence. Subsequently, the cells were exposed to different concentrations of silymarin (ranging from 10 to 100 μM) and incubated for 24 h in a CO2 incubator. Following this, 10 μl of MTT solution (5 μg/ml) was added to each well and the plates were kept in the dark for an additional 3 h. After removing the media, 100 μl of DMSO was added to dissolve the purple formazan crystals. The optical density (O.D.) value was measured at 570 nm using a microplate reader [16].

#### 2.2.4 Morphology analysis lung cancer A549 cell line

1×10^6^ cells were seeded into 6-well plates and allowed to grow until they reached approximately 70 % confluency. Subsequently, the cells were treated with silymarin compounds for 24 h. Following incubation, the cells were observed under an inverted light microscope (Euromex, Arnhem, The Netherlands) using a 20X objective lens, and morphological changes were documented.

#### 2.2.5 DAPI staining

After the silymarin treated, cells incubate with 4 μl of 10 mg/ml of DAPI staining. After 20 min incubation, wash the plate with 1X PBS to remove the excess staining. Visualized using Fluorescent microscope (Axiovert 5, Zeiss, Germany).

#### 2.2.6 Intracellular ROS determined by DCF-DA staining

After the silymarin treated, cells incubate with 4 μl of 10 mg/ml of DCF-DA staining. After 20 min incubation, wash the plate with 1X PBS to remove the excess staining. Visualized using Fluorescent microscope (Axiovert 5, Zeiss, Germany).

#### 2.2.7 Flow cytometry analysis

Initially, A549 cells were cultured in 6-well plates at a density of 1×10^6^ cells per well. Following the silymarin treatment period, cell cycle analysis and apoptosis assessment were analyzed according to the manufacturer's instructions using a BD FACS machine. The resulting data were then processed using Cell Quest Pro V 3.2.1 software (BD, USA).

#### 2.2.8 Evaluate the lipid peroxidation and enzymatic antioxidant assay

Cells were collected after a 24-hour treatment with silymarin and analysis the Lipid peroxidation level using Micro Lipid Peroxidation (MDA) Kit [CatLog No: KTB1050], evaluate the antioxidant enzymes, namely Superoxide Dismutase using a Micro Superoxide Dismutase (SOD) Activity assay Kit [CatLog No: KTB1030] and catalase using a Micro Catalase (CAT) Activity Assay Kit [CatLog No: KTB1040], all kits are procured from all kit from Abbkine, USA.

#### 2.2.9 Cell migration assay

A549 cells were plated in 6-well dishes at a concentration of 1*10^6^ cells per well. The cells were cultured until they attained 95 % confluency. A sterile 200 μl tip was used to create a scratch in the center of each well. The medium was then aspirated, and the wells were washed with phosphate-buffered saline (PBS). Afterwards, 1.5 ml of serum-free medium, either containing silymarin or not, was added to each well. An image of the wound areas was capture at initial and after 24 h incubation periods taken at 10X magnificence. Using Image J Software, measure the distance between wound area at initial time (t_0_) and after 24 h (t_24_), as a considered. The percentage of wound closure was calculated [17].

#### 2.2.10 Colony formation assay

1000 cells were seeded in each 60 mm petri dish, with regular media changes. After 3–4 days cells were exposed with or without silymarin, then allowed to grown for another 4 days. After incubation time, discard the medium and rinse with 1x PBS. Add 1 ml of ice-cold methanol to each 60 mm Petri dish for cells fixation, immediately placed in −20°C freezer. Then remove the methanol, add 1 ml of 0.5 % crystal violet and kept in dark condition for 30 min. After incubation, the cells were carefully rinsed with tap water to eliminate any surplus crystal violet. Then capture the image of 60 mm petri dish. Count the number of colonies using Image J software [18].

#### 2.2.11 Gelatin zymography

Gelatin zymography was used to analyze matrix metalloprotease-2, −9 (MMP-2) and (MMP-9). Following silymarin treatment, the medium was collected and kept at −20°C for later use. The experimental procedure was carried out as previously described by *Tajhya et al., 2017* [19].

### 2.3 Molecular docking

#### 2.3.1 Ligand and protein docking analysis

The research investigates the interactions between silymarin (CID: 5213), Doxorubicin (CID: 31703) and various proteins involved in cancer cell regulation. Specifically, the proteins MMP-2 (PDB ID: 7XJO) and MMP-9 (PDB ID: 1L6J) were analyzed, with their crystal structures sourced from the Protein Data Bank (PDB) (https://www.pdb.org/pdb). Docking were executed using the Lamarckian genetic algorithm (LGA) for up to 10 cycles, utilizing AutoDock software version 1.5.6. The resulting 3D and 2D docking structures were visualized and analyzed with Discovery Studio 2021.

### 2.4 Statistical analysis

All experiments were conducted in triplicate, and the results are presented as the mean ± standard deviation. The Student's t-test was applied to evaluate statistical significance, with a threshold of *p ≤ 0.05*, using GraphPad Prism software version 8.

## 3 Result

### 3.1 Prognostic makers of MMP-2, & MMP-9

**Fig. 1:**
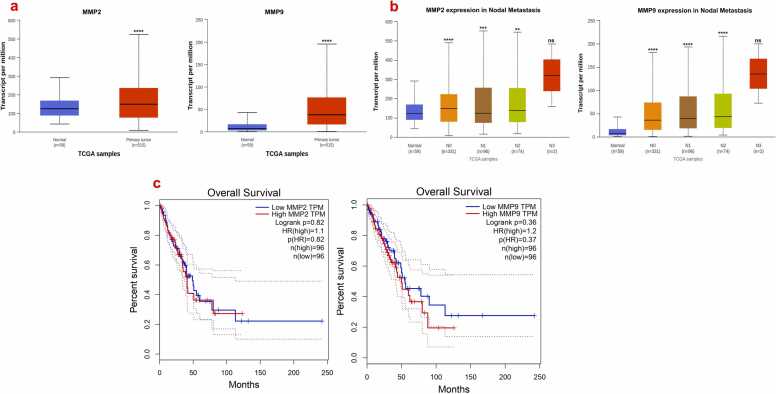
**a)** mRNA expression of MMP2 and MMP9 in normal vs lung cancer patients. **b)** Evaluate the MMP-2 and −9 expression in nodal metastasis (N0: No regional lymph node metastasis; N1: Metastases in 1–3 axillary lymph nodes; N2: Metastases in 4–9 axillary lymph nodes; N3: Metastases in 10 or more axillary lymph nodes). The data represented in mean ± S.D.’**’p<0.01, ‘***’ *p*< 0.001, ‘****’ p<0.0001, ns- non-significant as consider as a statistical significance **c)** Analysis the overall survival of MMP2 and MMP9 expression in lung cancer patient.

To validate the mRNA expression levels of matrix metalloprotease-2 (MMP-2) and matrix metalloprotease-9 (MMP-9) in both normal and lung cancer patients, we conducted a comparative analysis, as shown in Fig. 1(a). This analysis revealed significant differences in the expression of MMP-2 and MMP-9 between healthy individuals and those with lung cancer. Furthermore, we examined the expression of MMP-2 and MMP-9 across different nodal metastasis stages of lung cancer patients, as depicted in Fig. 1(b). The data shown that MMP-2 and MMP-9 expression levels increase with advancing nodal stages, highlighting their crucial roles in lung cancer metastasis and progression. The elevated expression of MMP-2 and MMP-9 was found to correlate with a more aggressive disease phenotype and poorer patient outcomes. Specifically, abnormal overexpression of these metalloproteases is associated with enhanced degradation of the extracellular matrix, facilitating tumor invasion and metastatic spread [20]. Consequently, this dysregulation contributes to a reduced survival rate among lung cancer patients, as illustrated in Fig. 1(c). These findings underscore the potential of MMP-2 and MMP-9 as prognostic markers and therapeutic targets in lung cancer management.

### 3.2 Cytotoxicity effect of Silymarin in Lung cancer A549 cell line

**Fig. 2:**
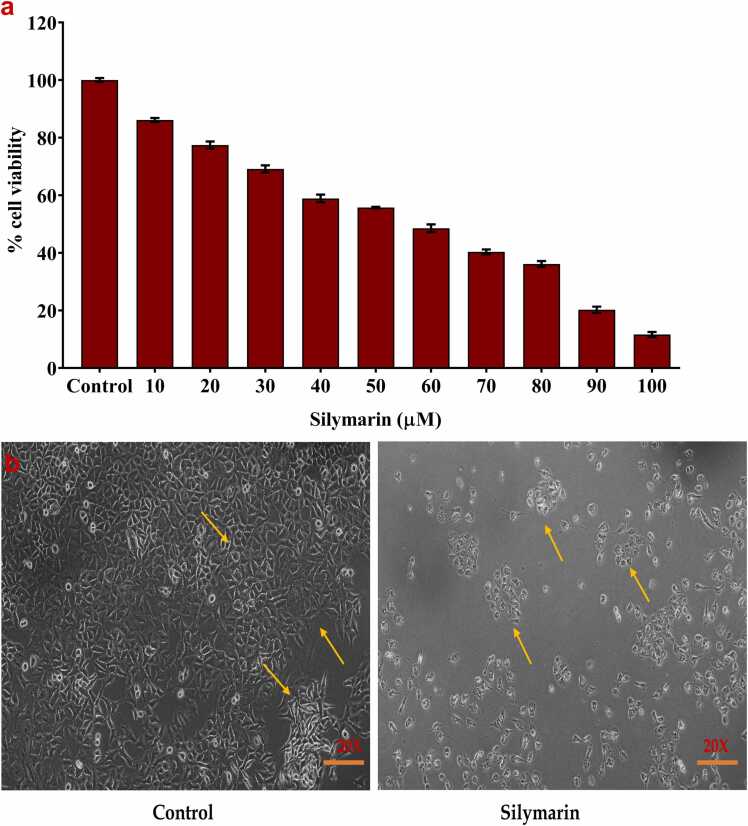
**a)** MTT assay was assessed to evaluate the efficiency of silymarin (0–100 µM) for 24 h of treatment. The data are represented in mean ± S.D. IC_50_ value was determined using Graph pad prism 8 software. Ic50 value were determined at 58 μM **b)** A549 morphological images (20x magnification) compared with control and silymarin treated for 24 hrs time intervals. Arrow mark indicate the A549 cell morphology changes.

The effect of Silymarin on cell proliferation was assessed using the MTT assay. Silymarin significantly inhibited the proliferation of A549 cells, with the inhibitory effect becoming evident after 24 h of incubation. Fig. 2(a) illustrates the variations in cell viability percentages between the control and Silymarin-treated cells. A concentration of 58 μM/ml of silymarin resulted in 50 % cell viability. Consequently, this concentration was used in subsequent experiments. Fig. 2(b) indicate the silymarin treated group shows the reduced cells population and altered A549 cell Morphology, compared with untreated groups.

### 3.3 Silymarin induce apoptosis in lung cancer A549 cells

**Fig. 3:**
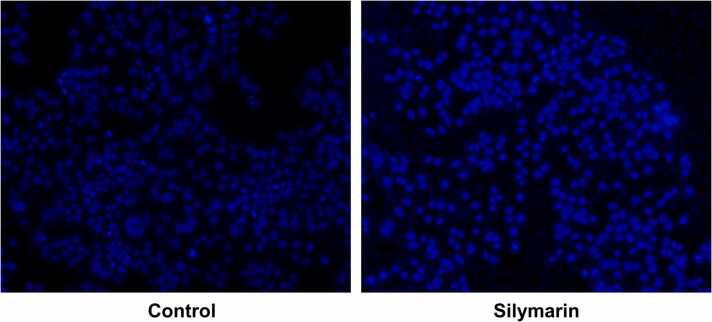
Fluorescent microscopic image of DAPI staining. Scale bar @50μm.

Fig. 3 indicates a significantly higher DAPI fluorescence intensity in the silymarin-treated group compared to the control group, suggesting increased chromatin condensation. The treated group showed irregular nuclear morphology and signs of apoptosis, while the untreated group had uniform, intact nuclei. These results suggest that silymarin induces apoptosis and alters nuclear integrity in treated cells.

### 3.4 Intra cellular ROS evaluated by DCF-DA staining

**Fig. 4:**
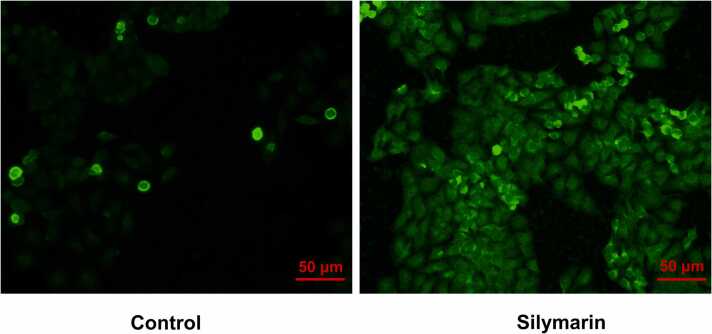
Fluorescent microscopic image of DCF-DA staining for evaluate the silymarin induce Intracellular ROS level in lung cancer A549 cell line. Scale bar @ 50μm.

The effect of silymarin on the generation of intracellular reactive oxygen species (ROS) in A549 lung cancer cells was evaluated using DCF-DA (2′,7′-dichlorofluorescin diacetate) staining in Fig. 4. The A549 cells were treated with silymarin, and the intracellular ROS levels were assessed by measuring the fluorescence intensity of DCF, the oxidized product of DCF-DA.

### 3.5 Silymarin arrest the cell cycle

**Fig. 5:**
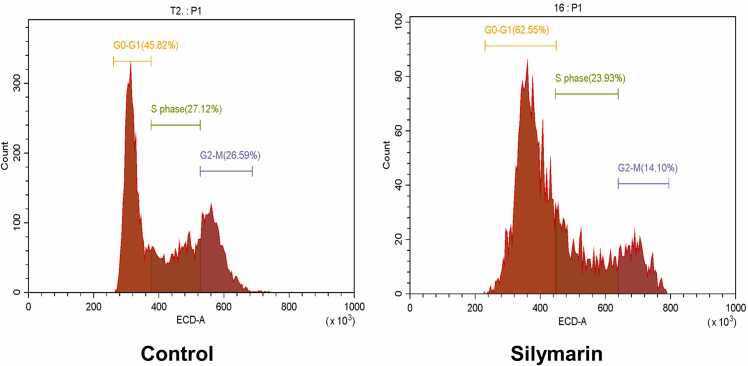
Silymarin induces cell cycle arrest in lung cancer A549 cells evaluated by Propidium Iodine (PI) by flow cytometry.

We perform the cell cycle analysis in A549 cells through flow cytometry. Our findings indicated that a 24-hour treatment with silymarin caused cell cycle arrest in the G0/G1 phase, increasing the cell population in this phase to 62.55 %, compared to 45.82 % in the untreated group, as depicted in Fig. 5.

### 3.6 Silymarin induce apoptosis in Lung cancer A549 cells

**Fig. 6:**
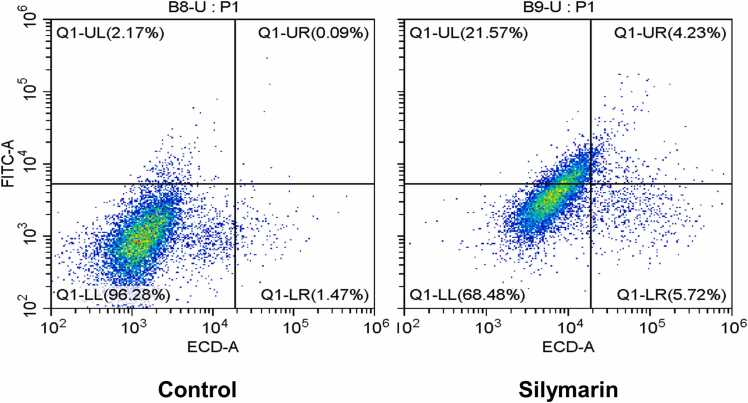
A549 cells were exposed to silymarin and analyzed for apoptosis by Annexin - FITC by flow cytometry. (LL-Live cells; UL-early apoptosis; UR-Late apoptosis; LR-Necrosis).

An Annexin V-FITC dual staining assay was performed to assess silymarin-induced apoptosis in A549 cells depicted in Fig. 6. This assay revealed a substantial rise in cell death after 24 h of treatment, with an apoptotic rate of 25.8 % and necrotic cell death at 5.72 %, as shown in Fig. 6. These results demonstrate silymarin's significant anticancer effects by promoting apoptosis in A549 cancer cells.

### 3.7 Silymarin induce the Lipid peroxidation and altered in Antioxidant enzymes activity

**Fig. 7:**
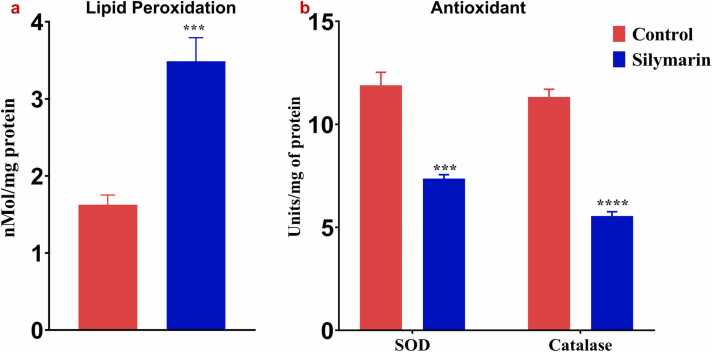
**a)** Silymarin enhanced the lipid peroxidation in lung cancer A549 cells. **b)** silymarin reduced the enzymatic antioxidant activity in lung cancer A549 cells. The data are represented as mean ± SD from three independent experiment. Statically different as denotes *** *p*< 0.001, ****p<0.0001.

To assess the level of lipid peroxidation by measuring malondialdehyde (MDA) in both control and silymarin-treated A549 lung cancer cells. As illustrated in Fig. 7(a), treatment with silymarin led to a notable increase in lipid peroxidation, with MDA levels rising to 3.4 nM/mg protein, indicating heightened oxidative stress in the cells. Furthermore, the activities of enzymatic antioxidants such as superoxide dismutase (SOD) and catalase (CAT) were evaluated, as shown in Fig. 7(b). Silymarin treatment (58 μM/ml) significantly reduced the activities of both SOD and CAT in A549 lung cancer cells.

### 3.8 Silymarin inhibit the A549 cell metastasis

**Fig. 8:**
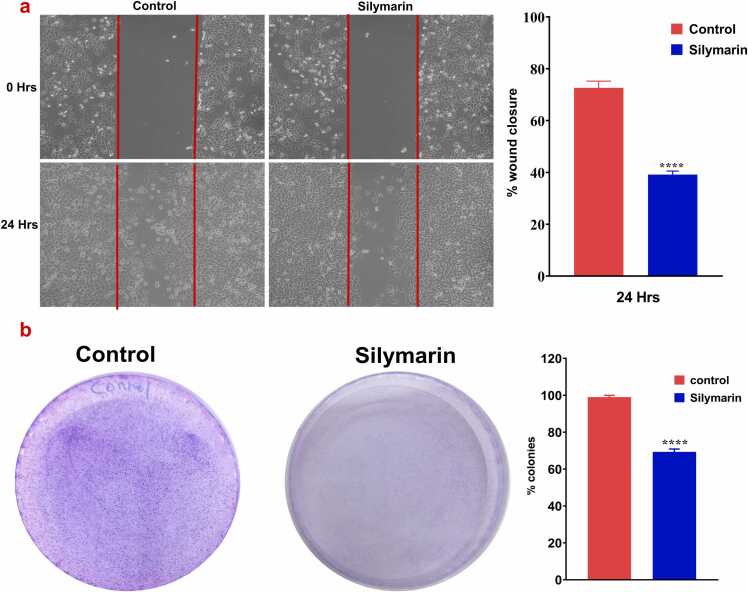
**a)** Cell migration assay. The cells were treated with silymarin for 24 h. Images were capture at 20X magnification. **b)** colony formation assay: Silymarin inhibit the A549 cell colony formation. The data are represented in mean ± S.D from three independent experiment. ****p<0.0001 denotes the statistical significance.

The cell migration assay was investigated to assess the impact of silymarin on the migratory capabilities of A549 lung cancer cells. As shown in Fig. 8(a), the wound area in the silymarin-treated group exhibited a significant reduction in wound closure, with only 40 % closure after 24 h compared to the control group. Additionally, A colony formation assay was conducted to evaluate the inhibitory effect of silymarin on cell proliferation. Fig. 8(b) demonstrates that silymarin-treated cells formed significantly fewer and smaller colonies compared to untreated cells, indicating that silymarin markedly inhibits colony formation, with the silymarin-treated group showing only 69 % of the colonies formed vs the control group.

### 3.9 Silymarin Inhibit the MMPs in Lung cancer A549 cell line

**Fig. 9:**
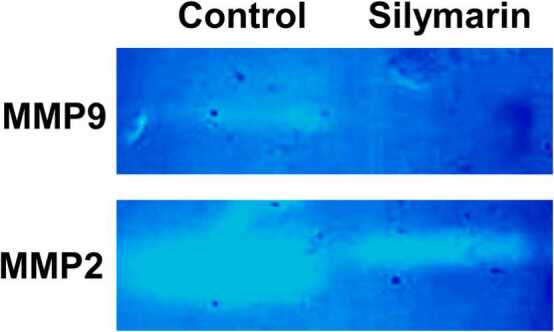
Analysis of matrix metalloproteinase 2 & 9 in control and silymarin treated groups.

The activity levels of matrix metalloproteases MMP-2 and MMP-9 were analyzed using gelatin zymography to determine the effect of silymarin on these key enzymes involved in metastasis. The results revealed that silymarin-treated groups exhibited significantly reduced band intensity for both MMP-2 and MMP-9 compared to untreated cells depicted in Fig. 9.

### 3.10 Molecular interaction between silymarin and targeted genes

**Fig. 10:**
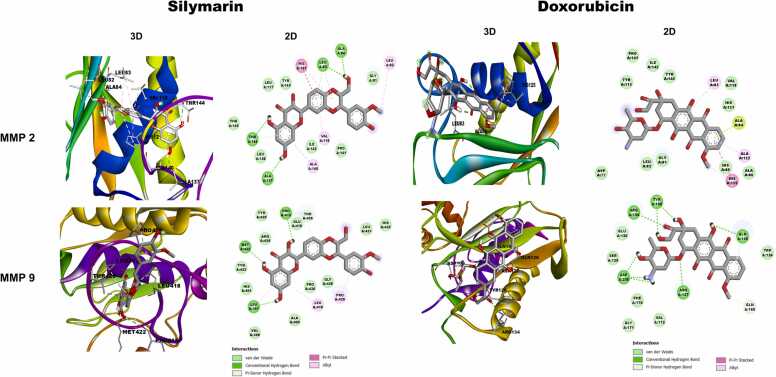
Molecular Docking analysis between silymarin, Doxorubicin and target proteins. 3D, & 2D visualization of molecular interaction of silymarin with targets proteins (MMP2 and MMP9).

In this study, molecular docking techniques were utilized to explore the binding interactions between silymarin and two significant matrix metalloproteinases (MMPs), MMP-2 and MMP-9, which are integral to cancer metastasis. The docking results indicated that silymarin has a high binding affinity for both MMP-2 and MMP-9, with binding energies of −10.26 kcal/mol and −6.69 kcal/mol, respectively shown in Table 1. AutoDockTools version 1.5.6 was used for the molecular docking, and the results were visualized using Discovery Studio 2021. The detailed binding interactions are depicted in Fig. 10. For MMP-9, silymarin forms three hydrogen bonds with the amino acid residues LEU397, MET422, and PRO415. For MMP-2, silymarin establishes four hydrogen bonds with THR144, ALA84, ALA137, and LEU83. Doxorubicin interact with MMPs with forming a 1 & 4 Hydrogen bonding with MMP2 and MMP9. These hydrogen bonds are critical as they contribute significantly to the stability and specificity of the ligand-protein complex.

**Table 1:** Binding energy of Silymarin and Doxorubicin with MMPs: Silymarin binds with MMP2 and MMP9, showing higher binding energies of −10.26 and −6.69 kcal/mol, respectively, compared to doxorubicin. Silymarin forms 4 and 3 hydrogen bonds with MMP2 and MMP9, while doxorubicin forms 1 and 4 hydrogen bonds with MMP2 and MMP9, respectively.

**Ligand**	**Protein**	**Hydrogen Bond Involved**	**Number of Hydrogen Bond**	**Bind Energy (Kcal/mol)**
Silymarin (CID: 5213)	MMP2 (7XJO)	THR144, ALA84, ALA137, & LEU83	4	− 10.26
	MMP9 (1L6J)	LEU397, MET422, & PRO415,	3	− 6.69
Doxorubicin (CID: 31703)	MMP2 (7XJO)	THR143	1	− 7.16
	MMP9 (1L6J)	TYR128, ASP206, GLN126, & ASN127	4	− 6.79

## 4 Discussion

Lung cancer is a prevalent malignancy affecting the large lung epithelial cells, ranking as the second most common cancer globally. It accounts for a significant number of cancer-related deaths, ranking as the fourth leading cause [21]. Extensive research over the past few decades have contributed to a comprehensive understanding of lung, making it one of the most well-studied cancers. Our study's findings highlight silymarin's potential as a potent inhibitor of MMP-2 and MMP-9 in A549 lung cancer cells. From the results, matrix metalloprotease −2 and −9 has shown the poor survival rate in lung cancer patient with its influence in lung cancer metastasis. Subsequently, we investigated cell viability assay of silymarin against the human lung cancer A549 cell line using the MTT assay method. The increased concentration of silymarin resulted in decreased viability in the lung cancer A549 cells and determining its IC_50_ value at 58 μM. Additionally, we observed and compared the control and treated groups of cell morphology, in the control groups, all cells have well-defined morphology in pebble like shape, but silymarin treated groups, shows the cells are displayed apoptosis hallmarks. The characteristics of apoptosis include condensed nuclei, cell shrinkage, membrane blebbing, apoptotic bodies, and echinoid spikes [22]. When cells undergo shrinkage, they lose their cell-cell interactions as their morphology collapses into round shapes, which leads to a loss of cell adhesion to the basal attachment [23]. In the experimental groups, a higher proportion of cells exhibited floating behavior in contrast to the control cells. As a result, findings from both MTT assays and morphology examinations suggest that the phytocompounds of silymarin possess the ability to impede cell proliferation and trigger cell apoptosis in lung cancer A549 cells. It is plausible that silymarin, characterized as a polyphenol compound, functions as a prooxidant, leading to the disruption of mitochondrial dehydrogenase activity [24]. This could explain the higher cytotoxicity observed at increased doses. A previous study corroborates this finding, showing that silibinin inhibits oral cancer cells (Ca9–22) and YD10B in a manner dependent on both time and concentration [25]. Another study *Kim et al., 2019*, revealed that silymarin inhibit the cell proliferation and shows the morphology changes in AGS human gastric cancer cells [26].

Antioxidant and prooxidant properties are crucial in the biochemical behavior of many polyphenolic compounds, including silymarin. While antioxidants are widely known for their ability to neutralize harmful free radicals, prooxidants can trigger oxidative stress by increasing reactive oxygen species (ROS) levels. This dual functionality is particularly intriguing in cancer treatment, where prooxidant activity can be strategically harnessed to selectively target cancer cells. In our research, we focused on the prooxidant activity of silymarin as a potential mechanism for its anticancer effects. The prooxidant activity of polyphenolic compounds has been substantiated by numerous studies [27]. Specifically, phenolic compounds ability to elevate ROS levels within cancer cells stands out as a critical factor in its anticancer efficacy [28], [29]. This sequence of reactions likely explains the increased ROS production observed during silymarin treatment. First, phenolic molecules oxidize to generate semiquinones, which are reactive intermediates. This is the start of the process. An autocatalytic cycle is started when these semiquinones combine with molecular oxygen to form superoxide anions (O_2_^•-^). This cycle produces hydrogen peroxide (H_2_O_2_) and semiquinones by continually oxidizing the phenolic compounds with superoxide anions. Furthermore, peroxidases found in cancer cells aid in the phenols' one-electron oxidation, which produces phenoxyl radicals. The quick oxidation of NADH to NAD^•^ by these phenoxyl radicals converts oxygen molecules to superoxide anions [30], [31]. Silymarin's strong anticancer action is supported by a chain reaction of reactions that account for the notable rise in ROS generation seen following treatment. Our study observed a significant rise in lipid peroxidation markers in silymarin-treated cancer cells. The well-documented prooxidant influence of hydroxycinnamic acids on DNA damage and lipid peroxidation is especially pronounced in the presence of Cu (II) ions [32]. Antioxidant enzymes play a crucial role in defending against tumor-promoting agents [33]. Notably, the progression of cells towards malignancy is frequently linked with a decline in the functioning of antioxidant enzymes like superoxide dismutase (SOD) and catalase (CAT), rendering the cells more vulnerable to prooxidant compounds [34]. Elevated levels of reactive oxygen species (ROS) inflict damage on DNA molecules within cancer cells. Upon detection of DNA damage, the tumor suppressor gene P53 becomes activated, prompting cell cycle arrest and initiation of DNA repair mechanisms. In situations where DNA damage is extensive, p53 induces programmed cell death, typically through apoptosis, via specific signaling pathways involving proteins such as Bax and repression of BCL2 [35]. Similar study was conducted in Cardamonin [36] and Epigallocatechin 3 gallate (EGCG) [37] induce the G2/M phase and induce caspase mediate cell death in breast cancer. According to *Chen et al., 2023*, demonstrate that Isolinderalactone induce the cell cycle arrest and apoptosis mediate ROS in colorectal cancer [38]. *Moreira et al., 2019*, revealed prooxidant celastrol demonstrate the ROS mediate Cell cycle arrest at G0/G1 phase and induced apoptosis in human colon cancer [39]. *Lee et al., 2021*, Picropodophyllotoxin have potential to trigger ROS mediate Cell cycle arrest at G1 phase and induce the apoptosis in human colon cancer [40]. Similarlly, our study found that the activities of antioxidant enzymes, specifically SOD and CAT, were reduced, while increased lipid peroxidation in silymarin-treated cancer cells. The excessive accumulation of ROS can cause DNA damage, which in turn triggers cell cycle arrest and apoptosis, as evidenced in Fig. 5, Fig. 6, are respectively.

The interaction between cancer cells and the extracellular matrix (ECM) represents a pivotal initial step in promoting various cellular processes such as migration, proliferation, and degradation. Matrix metalloproteinases (MMPs), a family of zinc-dependent endopeptidases, play a crucial role in ECM degradation, facilitating tumor cell invasion into surrounding tissues and dissemination through the bloodstream to distant locations [41]. Specifically, MMP-2 and MMP-9 are pivotal in degrading basement membrane type IV collagen, thereby promoting tumor growth, invasion, and angiogenesis. Elevated expression of MMP-2 is closely associated with tumor invasion, angiogenesis, metastasis, and recurrence, suggesting its significance as a therapeutic target in combating aggressive and metastatic tumor phenotypes [42]. Notably, MMP-2 and MMP-9 are considered prime targets for anticancer drug development due to their role in ECM degradation. Studies have demonstrated that curcumin exhibits inhibitory effects on cancer invasion and migration in A549 cells, potentially through the modulation of MMP activity. [43]. Similarly, previous reported by *Zhao et al., 2018*, indicate the resveratrol inhibit the cell migration, invasion and colony formation through modulation of MMPs, Akt and ERK1/2 in renal cell carcinoma cells [44]. *Chen et al., 2021*, demonstrate the Nicardipine potentially modulate MMP-9 to supress the Breast Cancer Migration [45]. Similarly, our study exhibit that silymarin have potential inhibition of MMP-2 and −9 in lung cancer A549 to hinder the Lung cancer migration.

## 5 Conclusion

The results of this study suggest that silymarin induces cancer cell death by reducing cell proliferation and antioxidant levels, while elevating intracellular ROS, lipid peroxidation, and triggering cell cycle arrest and apoptosis in the A549 Lung cancer cell line. Additionally, silymarin inhibits colony formation and the metastasis of lung cancer by modulating matrix metalloprotease −2 and −9. These results underscore silymarin's potential as an anticancer agent, particularly in inhibiting lung cancer metastasis and progression.

## Animal ethics

Not Applicable

## Author statements

There is no Conflict of Interest. All author are approve the revised manuscript.

## Funding

The authors Dr. Saravanan Muthupandian and Aruchamy Mohanprasanth acknowledge the ICMR (Ref. No. 2022-18903), Govt. of India for financial support. The authors acknowledge and extend their appreciation to the Researchers Supporting Project Number (RSP2024R124), 10.13039/501100002383King Saud University, Riyadh, Saudi Arabia.

## Declaration

None

## CRediT authorship contribution statement

**Mohanprasanth Aruchamy:** Writing – review & editing, Writing – original draft, Validation, Software, Methodology, Investigation, Formal analysis, Data curation, Conceptualization. **Srithika Srinivasan:** Software, Formal analysis, Data curation. **Muthupandian Saravanan:** Writing – review & editing, Visualization, Validation, Supervision, Project administration, Investigation, Formal analysis, Conceptualization. **Ahmed Nadeem:** Writing – review & editing, Validation, Data curation.

## Declaration of Competing Interest

The authors declare that they have no known competing financial interests or personal relationships that could have appeared to influence the work reported in this paper.

## Data Availability

Data will be made available on request.
